# Highly efficient and ultra-broadband graphene oxide ultrathin lenses with three-dimensional subwavelength focusing

**DOI:** 10.1038/ncomms9433

**Published:** 2015-09-22

**Authors:** Xiaorui Zheng, Baohua Jia, Han Lin, Ling Qiu, Dan Li, Min Gu

**Affiliations:** 1 Centre for Micro-Photonics in Faculty of Science, Engineering and Technology at Swinburne University of Technology, PO Box 218, Hawthorn, Victoria 3122, Australia; 2 Department of Materials Science and Engineering, Monash University, Clayton, Victoria 3800, Australia

## Abstract

Nanometric flat lenses with three-dimensional subwavelength focusing are indispensable in miniaturized optical systems. However, they are fundamentally challenging to achieve because of the difficulties in accurately controlling the optical wavefront by a film with nanometric thickness. Based on the unique and giant refractive index and absorption modulations of the sprayable graphene oxide thin film during its laser reduction process, we demonstrate a graphene oxide ultrathin (∼200 nm) flat lens that shows far-field three-dimensional subwavelength focusing (*λ*^3^/5) with an absolute focusing efficiency of >32% for a broad wavelength range from 400 to 1,500 nm. Our flexible graphene oxide lenses are mechanically robust and maintain excellent focusing properties under high stress. The simple and scalable fabrication approach enables wide potential applications in on-chip nanophotonics. The wavefront shaping concept opens up new avenues for easily accessible, highly precise and efficient optical beam manipulations with a flexible and integratable planar graphene oxide ultrathin film.

## Abstract (web summary)

Lenses made from metamaterials often suffer from narrow operational bandwidth and complex design. Here, Zheng *et al.* demonstrate an ultrathin graphene oxide lens with farfield three-dimensional subwavelength focusing from the visible to near infrared and an absolute focusing efficiency greater than 32%.

## Graphical abstract

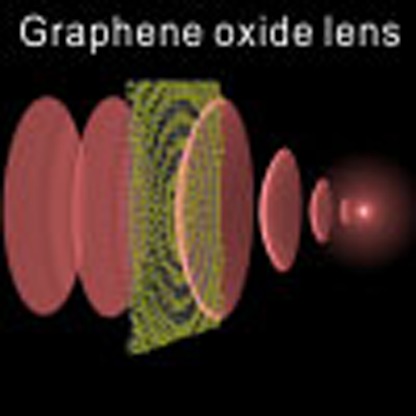

The optical lenses are indispensable components in almost all aspects of science and technology including imaging, sensing, communications, medical diagnosis and treatment^1^,^2^,^3^. Recently, the rapid development in nano-optics and on-chip photonic systems has placed stringent demand for ultrathin flat lenses with three-dimensional (3D) subwavelength focusing capability^4^,^5^,^6^. Conventional optical lenses rely on sufficient phase accumulation when a beam propagates over optical paths much larger than the light wavelength to realize the desired wavefronts^7^. The phase modulation *ϕ* of a conventional lens can be expressed as *ϕ*=Δ*n* × |**k**| × Δ*t*, where Δ*n* is the refractive index difference between the lens and the surrounding medium, **k** (|**k**|=2*π*/*λ*) is the wave vector of light and Δ*t* is the thickness of the lens. Owing to the limited refractive index difference (Δ*n*) of naturally available transparent dielectrics compared with the surrounding medium (mostly air), conventional geometrical lenses are fundamentally incapable of achieving subwavelength-scale (Δ*t*<*λ*) ultrathin flat lenses with decent 3D diffraction-limited focusing^8^.

The recent breakthroughs in nanophotonics have led to the design and demonstration of a number of ultrathin flat lens concepts^2^,^5^,^6^,^9^,^10^,^11^,^12^,^13^,^14^, such as metamaterials^12^, metasurfaces^13^ or super-oscillations^14^, which have achieved subwavelength resolution in two dimensions. In particular, all-dielectric metasurface lenses show promising results in reducing the Ohmic losses^15^,^16^. However, the narrow operational bandwidth, the complex design and time-consuming multi-step manufacturing process have greatly limited their real-life applications especially when large-scale production and micro-devices integration are required. Therefore, it is highly desirable to develop ultrathin flat lenses with 3D subwavelength focusing capability, a large operational bandwidth, and a practically useful focusing efficiency (>20%) with a simple and low-cost fabrication method.

Here, we propose a new ultrathin flat lens concept that is able to effectively manipulate the phase and amplitude of an incident beam simultaneously. We report on a 200-nm-thick graphene oxide (GO) flat lens with 3D subwavelength focusing that is able to tightly focus broadband light from the visible to near infrared (VIS–NIR; ∼1,100 nm bandwidth) with an averaged absolute focusing efficiency of >32% over the entire band.

## Results

### Conceptual design of the GO lens

**Figure 1:**
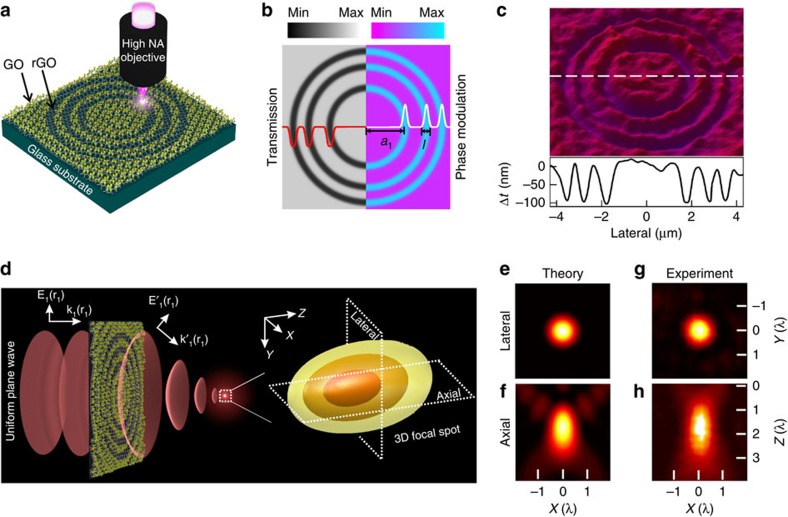
The design of the GO lens. (**a**) Conceptual design and laser fabrication of the GO ultrathin lens. (**b**) Amplitude and phase modulations provided by the transmission and refractive index difference, respectively, between the GO and rGO zones. (**c**) Topographic profile of the GO lens measured with an optical profiler. (**d**) Left: Schematic of the wavefront manipulation by the GO lens converting the incident plane wave into a spherical wavefront. Right: Intensity distributions of the 3D focal spot predicted by the analytical model for a GO lens (*a*_1_=1.8 μm, *N*=3). (**e**,**f**) Theoretical focal intensity distributions in the lateral and axial directions. (**g**,**h**) Experimental focal intensity distributions along the lateral and axial directions.

The conceptual design of the GO ultrathin flat lens and the corresponding theoretical and experimental results are illustrated in Fig. 1. The GO flat lens is made possible by the submicrometre concentric ring fabrication in a sprayed GO film by using the mask-free direct laser writing (DLW) method to covert the GO into reduced GO (rGO) via the photoreduction process, as illustrated in Fig. 1a. The laser power controlled removal of the oxygen groups in the exposed regions of the GO film leads to three continuously tunable local physical property variations: the reduction of film thickness, the increase of refractive index and the decrease of transmission/increase of extinction coefficient (Supplementary Figs 1 to 4)^17^. As a result, the three tunable local property variations provide an unprecedented flexibility in designing new ultrathin flat lens concept with capabilities of both amplitude and phase control (Fig. 1b), and thus make our GO flat lens concept fundamentally different from the conventional Fresnel lenses and other flat lenses, which mostly rely on either phase or amplitude modulation solely^7^,^18^.

To elucidate the light interaction with the GO flat lens, an analytical model based on the Rayleigh–Sommerfeld diffraction theory^7^ is developed to evaluate the focusing capability of the GO lens. When a uniform plane wave impinges on the GO lens, part of the beam is absorbed and refracted by the rGO zones, experiencing substantial amplitude as well as phase modulations. The other part of the beam propagating through the GO zones only experiences ignorable amplitude modulations. The 3D focusing is a result of the interferences of wavelets originated in the lens plane from different zones^19^, as illustrated in Fig. 1d. It should be emphasized that although the refractive index modulation from GO to rGO is giant (Δ*n*∼0.8, one to two orders of magnitude larger than conventional refractive materials^20^), the resultant maximum phase change between adjacent GO and rGO zones is smaller than π, which is the required phase change for effective constructive interference in the focal region^7^. Under such a circumstance, the amplitude modulation in the rGO zones can make a substantial contribution to the final well-defined 3D focusing (Supplementary Figs 5 to 7 and Supplementary Note). The positions of each ring hold the key to allow both the amplitude and phase modulations positively contribute to the constructive interference in the focal region to generate a focal spot in the far field. To achieve constructive interference of all the GO and rGO zones in the focal region, the radius of the *m*th rGO zone, *a*_m_, needs to satisfy the following condition (Supplementary Note 1):


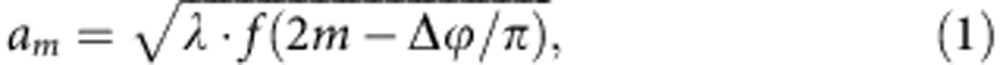


where *f* is the designed focal length of the GO flat lens and Δ*ϕ* is the phase modulation between GO and rGO zones. It is thus evident that 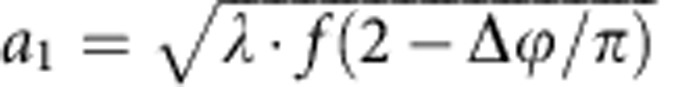 determines the positions of the rest rGO zones. As a result, we use *a*_1_ and the total rGO zone number (*N*) to define the geometry of our GO flat lenses in this paper.

To achieve the optimized diffraction efficiency in the focal region, Gaussian profiles for the phase and amplitude modulation functions (Fig. 1b,c), which can be naturally facilitated by the intensity profile of the fabrication laser (Supplementary Fig. 8), are employed to effectively direct the majority of the incident light to the first diffraction order^21^. In this way, the theoretical maximal focusing efficiency of 50% can be achieved. In contrast, the conventional rectangular profiles distribute the light energy to a series of diffraction orders that severely degrade the focusing efficiency and resolution of the main focal spot (Supplementary Fig. 9).

### Experimental characterizations of the GO lenses

To validate the theoretical model, the dependence of the focusing intensity of the GO lenses on *a*_1_ is investigated. The calculated focusing intensity presents a nonlinear dependence on *a*_1_, reaching a maximum for a three-ring (*N*=3) GO lens when *a*_1_=1.8 μm. And the experimentally measured dependence of the normalized focusing intensities on *a*_1_ agrees remarkably well with the theoretical prediction (Supplementary Fig. 10). The calculated 3D focal intensity distributions for the optimized lens (*a*_1_=1.8 μm) is shown in Fig. 1d, with the cross-sectional plots in the lateral and axial directions shown in Fig 1e,f, respectively. Well-defined strong focus with ignorable side-lobes (<10% of the intensity of the central lobe) is observed. The full-width at half-maximum (FWHM) of the focal spot in the lateral direction (FWHM_lateral_) is 0.8*λ* and that in the axial direction (FWHM_axial_) is 1.6*λ*, leading to a high-quality 3D focal spot with wavelength-scale resolution. The corresponding GO lens is experimentally fabricated with the surface profile shown in Fig. 1c, in which the Gaussian surface morphology is clearly evident. The measured focal intensity distributions in the lateral (Fig. 1g) and axial (Fig. 1h) directions under 700 nm light illumination almost reproduce the theoretical plots, with a measured FWHM_lateral_=567 nm (0.81*λ*) and a FWHM_axial_=1.3 μm (1.8*λ*). The slight difference between the experiment and theory might be due to the imperfection of the fabrication and the surface roughness of the GO film.

Although 3D wavelength-level focusing has been demonstrated for the ultrathin GO flat lens, it is not the best focusing resolution that the GO lens can achieve. According to our theoretical predictions, increasing the ring number *N* (equivalent to increase the aperture of the lens) and/or reducing *a*_1_ can both improve the resolution (Supplementary Fig. 11). However, practically the maximum *N* and minimum *a*_1_ are both determined by the smallest feature size (*l*) that the laser can fabricate on the GO film. To separate two consecutive rings, the feature size has to be smaller than the distance between the two rings as *l*<*a*_m+1_−*a*_m_. Meanwhile, the distance between the two outmost rings determines the maximum convergence angle *β* of the lens as 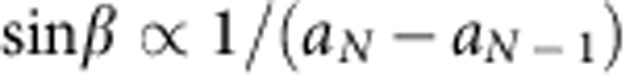. Therefore, the minimal feature size of each rGO zone is the key parameter practically limiting the achievable focusing resolution of the lens. However, laser fabrication based on thermal reduction in the literature can only achieve minimum feature size >1 μm (ref. 22), which severely restricts the lateral focusing resolution of the GO flat lens to be over 6.5*λ* according to our experiments using a MHz repetition rate laser (not shown). Recently, it has been reported that GO has a threshold photon energy for photochemical reduction at 3.2 eV (*λ*=390 nm)^23^. Therefore, for a high peak power femtosecond laser beam operating at 800 nm, it is potentially possible to harness the nonlinear photochemical mechanism to enhance the fabrication resolution^24^,^25^. As a result, to minimize the thermal effect and potentially make use of the high-resolution nonlinear reduction mechanism, we tightly focus low repetition rate, femtosecond pulsed, laser beam (100 fs, 10 kHz, 800 nm) with a high numerical aperture (1.4) objective to ensure the interval of the laser pulse to be sufficiently larger than the thermal diffusion constant of the GO^22^. As a result, a record small line width of ∼300 nm (0.38 *λ*) is achieved in the rGO laser fabrication (Supplementary Fig. 2), which represents a more than threefold resolution enhancement compared with the literature^22^. Consequently, maximum six rings with a minimum *a*_1_=730 nm can be fabricated. It should be noted that the notation *a*_1_ is the radius of the first ring of the lens, whereas the minimal feature size is the smallest fabricated line width *l*. The minimum *a*_1_ is determined by the minimum feature size *l* (∼300 nm).

**Figure 2:**
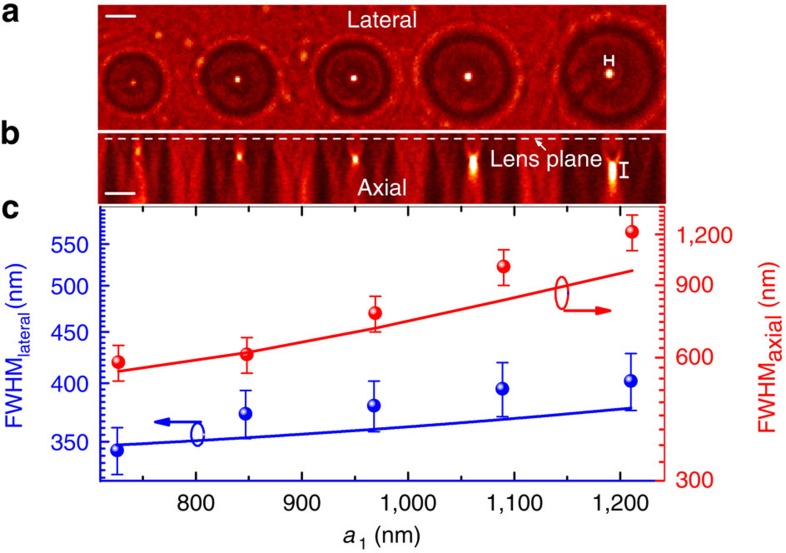
Dependence of focusing resolution on *a*_1_. Top: Measured cross-sectional intensity distributions in the lateral (**a**) and axial (**b**) directions, respectively. The lens plane is indicated as the white dash line in **b**. (**c**) The measured dependence of FWHM_lateral_ (blue spheres) and FWHM_axial_ (red spheres) on *a*_1_. The error bars on both FWHM_lateral_ and FWHM_axial_ are determined via resampling. The corresponding theoretical predictions (blue and red solid lines) are also presented and show excellent agreements with experimental results. Scale bar, 2 μm.

Figure 2 presents the measured lateral and axial focusing intensity distributions of the experimentally fabricated six-ring ultrathin GO flat lenses with different *a*_1_ from 730 to 1,200 nm. It is obvious that well-defined 3D foci are achieved in all cases. The FWHM in both the lateral and axial directions of the foci reduce as the decrease of *a*_1_ following exactly the same trend predicted by our theory (Fig. 2c). The highest lateral resolution is 343 nm (0.49*λ*) and axial resolution is 584 nm (0.83*λ*) illuminated by a light beam of *λ*=700 nm, leading to a 3D diffraction-limited resolution of *λ*^3^/5. The demonstrated 3D subwavelength focal spot is to our knowledge the best far-field 3D resolution achieved so far for an ultrathin flat lens.

### Focus intensity tuning and large-scale fabrication

**Figure 3:**
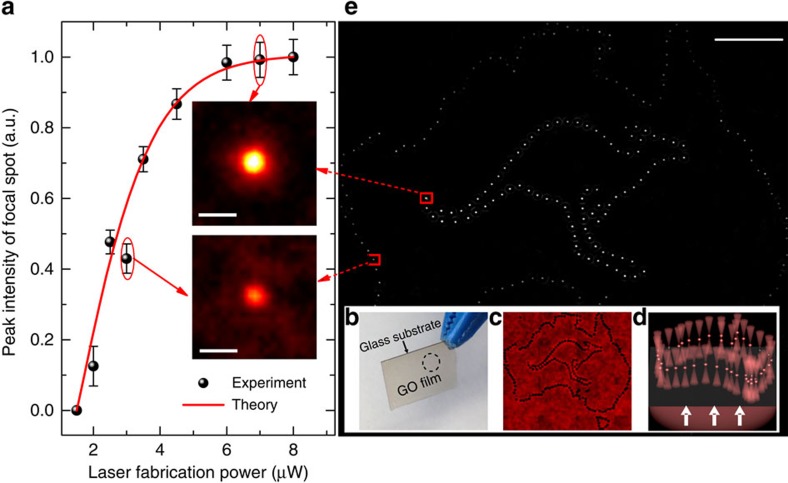
Laser controllable tunability of the focus intensity. (**a**) Normalized focusing intensities of GO lenses fabricated at increasing laser powers from 1.5 to 8 μW (black spheres). Theoretical prediction (red solid line) shows remarkable agreements with the experimental results. The error bar is based on the statistical average by sampling GO lenses with the identical fabrication power. Insets: Cross-sectional intensity distributions in the lateral direction of two GO lenses fabricated at 3 and 7 μW, respectively. Scale bar, 1 μm. (**b**) The prepared large size 200-nm-thick GO thin film on a glass substrate. (**c**) Two sets of GO lenses following the contours of an Australian map and a kangaroo are patterned in a 1.5 mm^2^ area (dashed circle in **b**). (**d**) Schematic diagram of focusing of the GO lenses under a plane wave illumination (white arrows). (**e**) Measured microscopic images of the contours of an Australian map and a kangaroo at the lens focusing plane showing different focusing intensities. Scale bar, 200 μm.

The fact that the phase and amplitude modulations of rGO zones are highly dependent on the fabrication laser power offers a simple and unique approach to tune the focus of the ultrathin GO flat lens. To demonstrate this capability, GO lenses with an identical geometry (*a*_1_=1.6 μm, *N*=3) are fabricated with increasing laser powers from 1.5 to 8 μW. The normalized peak focusing intensity is found to increase with the fabrication laser power monotonically, as shown in Fig. 3a. To further illustrate the laser controllable tunability of the focus intensity and large-scale fabrication capability, a large size 200-nm-thick GO thin film is prepared on a glass substrate (Fig. 3b), and a contour of Australian map comprising of 162 GO lenses and a contour of a kangaroo comprising of 89 GO lenses are fabricated with 3 and 7 μW laser powers, respectively, in a 1.5 mm^2^ area (dashed circle in Fig. 3b), as shown in the microscopic image in Fig. 3c. By shining light on the fabricated region (Fig. 3d), the kangaroo contour shows a much larger brightness compared with the contour of Australian map because of the higher focusing intensity of the comprising lenses (Fig. 3e). The enlarged lateral cross-sectional intensity distributions of GO lenses taken from the kangaroo and Australian map are also shown in the insets of Fig. 3a, which highlight the focusing intensity differences. These results demonstrate not only the versatile laser addressable tunability of the GO flat lenses, but also the scalable and reliable laser fabrication of GO films ready for large-scale manufacturing and applications.

### Broadband focusing

**Figure 4:**
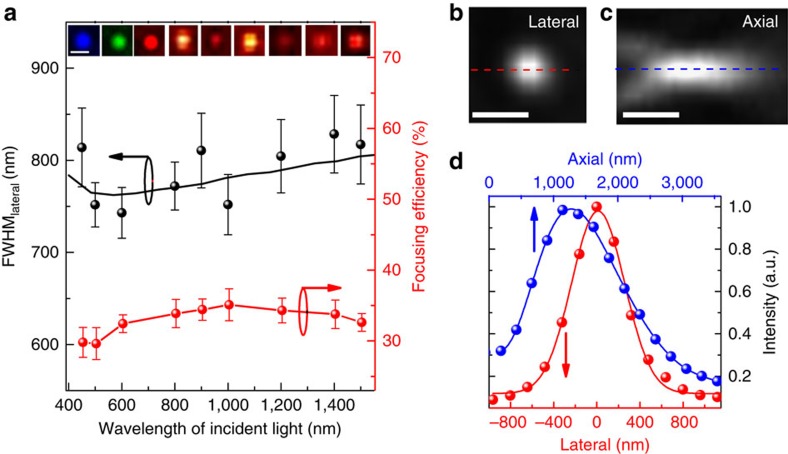
The broadband focusing capability from visible to infrared. (**a**) Characterization of the broadband focusing ability of a GO lens (*a*_1_=3.3 μm, *N*=3). Both FWHM_lateral_ (black spheres) and focusing efficiency (red spheres) are measured over a broadband illumination of the wavelengths from 450 to 1,500 nm. The error bars on both FWHM_lateral_ and efficiency are determined via resampling. Theoretical predictions of FWHM_lateral_ (black solid line) are also presented matching well with the experimental results. Insets: Cross-sectional intensity distributions along the lateral direction captured by two CCDs operating at the visible and infrared, respectively. Different colours indicating different input wavelengths schematically. Scale bar, 1 μm. (**b**–**d**) Focusing ability of a GO lens (*a*_1_=1.2 μm, *N*=3) under a white light illumination. Measured cross-sectional intensity distributions along the lateral (**b**) and axial (**c**) directions under a white light illumination. The intensity distributions along the red dashed line in **b** and blue dashed line in **c** are also plotted (red and blue spheres in **d**) and fitted (red and blue solid lines in **d**), respectively. Scale bar, 1 μm.

One of the critical challenges of the current ultrathin lenses, such as metalenses^2^, plasmonic lenses^9^ and super-oscillation lenses^14^, is the narrow bandwidth because of the constrains from both the dispersion properties of the constituting materials and the fundamental lens operation mechanisms^26^. The state-of-the-art bandwidth for an ultrathin flat lens is 280 nm (ref. 27), whereas most ultrathin flat lenses are only functional at a single wavelength^2^,^10^. In contrast, the wavefront shaping mechanism of our GO lens originates from the effective amplitude and phase modulations between the GO and rGO zones. Given the fact that the large modulations of both the refractive index and extinction coefficient between GO and rGO are almost maintained from visible (above 350 nm) to infrared regions (Supplementary Figs 3, 4 and 12), the GO lens provides a possible mechanism for achieving broadband focusing. To realize the broadband focusing, we design and fabricate a GO lens (*a*_1_=3.3 μm, *N*=3) with the optimal performance over the entire VIS–NIR wavelength range based on our theoretical guidance (Supplementary Fig. 13). The cross-sectional focusing intensity distributions in the lateral direction of this lens are experimentally characterized under different illumination wavelengths from 450 to 1,500 nm, as shown in the insets of Fig. 4a. The results clearly demonstrate that well-defined subwavelength foci have been achieved at all wavelengths. Moreover, the measured absolute focusing efficiencies, defined as the ratio of the power in the focal region to the total input power before the lens, are higher than 32% on average in the entire measured VIS–NIR band (Fig. 4a), representing a more than 30-time's improvement compared with the state-of-the-art ultrathin flat lens^13^. It should be emphasized that such a broadband, high-resolution focusing over 1,050 nm bandwidth is achieved with a single GO lens. In addition, it is interesting to note that the measured FWHM_lateral_ of the foci at different wavelengths shows an almost constant value of ∼0.8 μm, which is also predictable from the operating principle of our ultrathin lenses.

The broadband focusing of the GO lens allows us to minimize the chromatic aberration specifically in the visible regime, which is critical for multi-wavelength optical imaging and communications^28^. By carefully designing *a*_1_ of our GO lens, the shift of the focal length over all visible wavelengths can be smaller than the depth of focus, which suggests that our GO lens can be optimized to focus the incoherent white light within a common focal region. To achieve the minimal chromatic aberration in the visible regime, a GO lens (*a*_1_=1.2 μm, *N*=3) has been fabricated and characterized under white light illumination. The cross-sectional intensity distributions in both the lateral and axial directions are shown in Fig. 4b,c, exhibiting an obvious high-resolution 3D focal spot. By fitting the intensity distributions (Fig. 4d), the FWHM in the lateral and axial directions are found to be 577 nm and 1.56 μm, respectively, which even outperform most of the ultrathin lens operating under single wavelength illumination^2^,^10^. This is to our knowledge the first demonstration of a subwavelength-scale flat lens that can focus white light three-dimensionally with minimal chromatic aberration.

### Mechanical robustness and wavefront engineering of GO films

**Figure 5:**
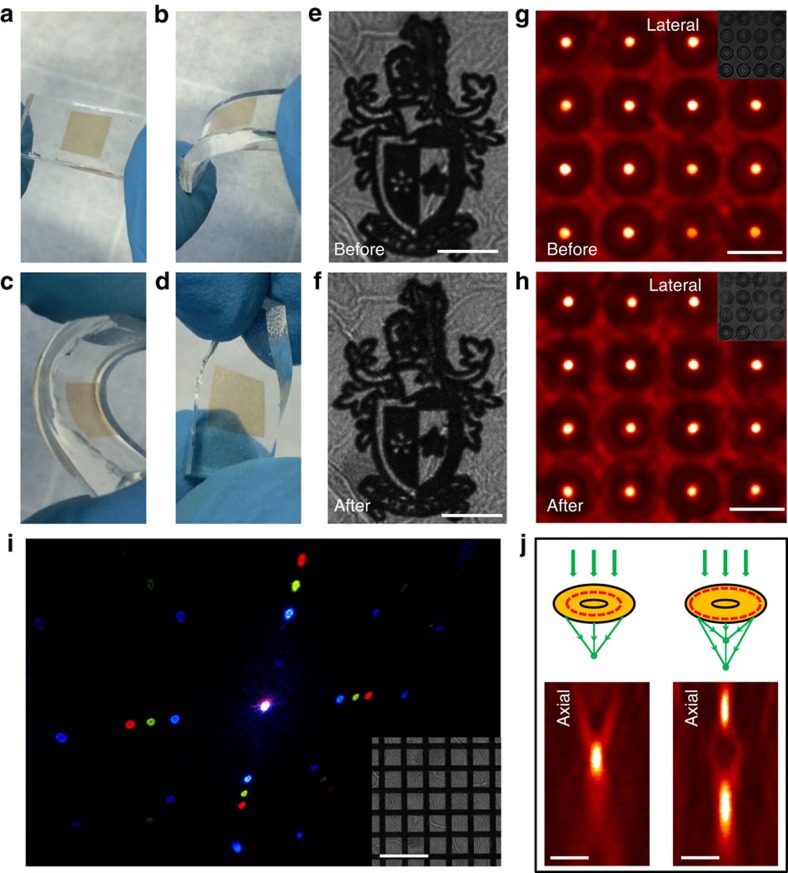
The flexibility and versatility in wavefront manipulations. (**a**–**d**) Bending and twisting of the GO thin film on a polydimethylsiloxane substrate. (**e**,**f**) Microscopic images of Swinburne logo fabricated on the GO thin film before (**e**) and after (**f**) bending. Scale bar, 10 μm. (**g**,**h**) Microscopic images of the GO lens array at the focusing plane before (**g**) and after (**h**) bending. Scale bar, 30 μm. Insets: microscopic images of the GO lens array fabricated on the GO film. (**i**) Diffraction image of a two-dimensional grating, retrieved by using three beams at wavelength of 405, 561 and 633 nm simultaneously. Inset: microscopic image of the two-dimensional grating fabricated on the GO film. Scale bar, 20 μm. (**j**) Comparison of the single focal spot (left column) and multi-foci GO lenses (right column). The designs are shown schematically (top panel) and the focal intensity distributions along the axial directions are characterized experimentally (bottom panel). Scale bar, 2 μm.

Given its strong mechanical robustness, large-scale integratability and simultaneous patterning and beam manipulation abilities during the one-step mask-free DLW process, GO ultrathin films have the potential to revolutionize the next-generation integrated optical systems by making miniaturized and fully flexible photonics devices. To illustrate its mechanical flexibility and the versatility in wavefront manipulations, a large-scale GO thin film is integrated on a flexible polydimethylsiloxane substrate and then is patterned with complex logos as well as other functional diffractive optical elements, as shown in Fig. 5. After various bending and twisting (Fig. 5a–d), both the complex logo (Fig. 5e,f) and the 4 × 4 GO lens array (Fig. 5g,h) survive without any compromise of the morphology or optical performance, demonstrating the excellent mechanical strength of our GO films. Moreover, the wavefront shaping mechanism with our ultrathin GO film is readily applicable to other diffractive optical element design. Figure 5i presents a two-dimensional grating, in which rGO (darker regions) and GO offer periodic phase and amplitude modulations. The diffraction pattern under 405, 561 and 633 nm lasers simultaneous illumination clearly shows the high diffraction performance of the GO grating. Finally, as shown in Fig. 5j, a multi-foci GO lens (right column) can be realized by simply adjusting the position of the rGO rings in the single focal spot GO lens design (left column), which offers a great potential for super-resolution imaging, optical data storage and sensing applications^29^.

## Discussion

The new wavefront shaping concept with laser patterned GO ultrathin films provides new and variable solutions for ultralight weight, highly efficient, highly integratable and flexible optical systems, opening up new avenues for various multidisciplinary applications including non-invasive 3D biomedical imaging, laser tweezing, all-optical broadband photonic chips, light harvesting, aerospace photonics, optical microelectromechanical systems and lab-on-chip devices.

## Methods

### GO film formation

The high-quality GO solutions are synthesized by the chemical reduction of graphite via a modified Hummers Method^30^. Then homogenous GO thin films of controllable thicknesses can be prepared by the spraying method with varied spraying speed and solution concentration.

### Femtosecond laser fabrication

A homemade DLW system is used to fabricate the GO lens on the as-prepared GO film directly^31^. To minimize the thermal effect, a low repetition rate, femtosecond pulsed, laser beam (100 fs pulse, 10 kHz, 800 nm) is used to reduce the GO film through the DLW method. The sample is mounted on a 3D nanometric piezo stage. A computer-controlled system is used to create 3D arbitrary structures.

### Optical characterization of GO films

A scanning electron microscope (RAITH150-TWO), an optical profiler (Bruker ContourGT InMotion), an ellipsometer (J.A. Woollam M-2000) and a spectrometer (PerkinElmer UV/Vis Spectrophotometer) have been used to characterize the morphology and optical parameters of the GO and rGO films.

### Imaging system and broadband measurement

A Nikon N-STORM microscope with 700 nm plane wave illumination is used for the visible lens characterization. The cross-sectional distributions of the generated focal spots of the GO lenses are captured with a numerical aperture=1.4, × 100 objective into a charge-coupled device (CCD) camera. By gradually adjusting the distance between the objective and the GO lens in a step of 100 nm, we are able to obtain the optical intensity distributions at different axial positions, and the 3D images of the focal spot can be reconstructed. By normalizing the sensitivity and exposure time of the CCD camera, the lateral cross-sectional intensity distributions can be captured and the peak focusing intensities can be compared directly.

For the broadband focusing characterization, a super-continuum laser with illumination wavelengths from 450 to 1,500 nm is used as the light source. Two CCD cameras, operating at visible (Watec 902H3 SUPREME) and infrared (Xenics Xeva-1.7-320) regions, respectively, are used to capture the focal plane images of the GO lens at different wavelengths.

## Additional information

**How to cite this article:** Zheng, X. *et al.* Highly efficient and ultra-broadband graphene oxide ultrathin lenses with three-dimensional subwavelength focusing. *Nat. Commun.* 6:8433 doi: 10.1038/ncomms9433 (2015).

## Supplementary Material

Supplementary Information Supplementary Figures 1-13, Supplementary Note 1 and Supplementary References

## References

[b1] Wang Z. *et al.* Optical virtual imaging at 50 nm lateral resolution with a white-light nanoscope. Nat. Commun. 2, 218 (2011). 21364557 10.1038/ncomms1211

[b2] Lu D. & Liu Z. Hyperlenses and metalenses for far-field super-resolution imaging. Nat. Commun. 3, 1205 (2012). 23149749 10.1038/ncomms2176

[b3] Hell S. W. Toward fluorescence nanoscopy. Nature Biotechnol. 21, 1347–1355 (2003). 14595362 10.1038/nbt895

[b4] Pendry J. B. Negative refractive makes a perfect lens. Phys. Rev. Lett. 85, 3966–3969 (2000). 11041972 10.1103/PhysRevLett.85.3966

[b5] Liu Z. *et al.* Focusing surface plasmons with a plasmonic lens. Nano Lett. 5, 1726–1729 (2005). 16159213 10.1021/nl051013j

[b6] Fang N., Lee H., Sun C. & Zhang X. Sub-diffraction-limited optical imaging with a silver superlens. Science 308, 534–537 (2005). 15845849 10.1126/science.1108759

[b7] Hecht E. Optics Addison-Wesley (2002).

[b8] Lohmann A. W. Scaling laws for lens systems. Appl. Opt. 28, 4996–4998 (1989). 20555989 10.1364/AO.28.004996

[b9] Verslegers L. *et al.* Planar lenses based on nanoscale slit arrays in a metallic film. Nano Lett. 9, 235–238 (2009). 19053795 10.1021/nl802830y

[b10] Yu N. & Capasso F. Flat optics with designer metasurfaces. Nat. Mater. 13, 139–150 (2014). 24452357 10.1038/nmat3839

[b11] Ni X., Ishii S., Kildishev A. V. & Shalaev V. Ultra-thin, planer, Babinet-inverted plasmonic metalenses. Light Sci. Appl. 2, e72 (2013).

[b12] Kildishev A. V., Boltasseva A. & Shalaev V. M. Planar photonics with metasurfaces. Science 339, 1232009 (2013). 23493714 10.1126/science.1232009

[b13] Aieta F. *et al.* Aberration-free ultrathin flat lenses and axicons at telecom wavelengths based on plasmonic metasurfaces. Nano Lett. 12, 4932–4936 (2012). 22894542 10.1021/nl302516v

[b14] Rogers E. T. F. *et al.* A super-oscillatory lens optical microscope for subwavelength imaging. Nat. Mater. 11, 432–435 (2012). 22447113 10.1038/nmat3280

[b15] West P. R. *et al.* All-dielectric subwavelength metasurface focusing lens. Opt. Express 22, 1593–1595 (2014). 10.1364/OE.22.026212 25401653

[b16] Lin D. *et al.* Dielectric gradient metasurface optical elements. Science 345, 298–302 (2014). 25035488 10.1126/science.1253213

[b17] Zheng X., Jia B., Chen X. & Gu M. *In situ* third-order nonlinear responses during laser reduction of graphene oxide thin films towards on-chip nonlinear photonic devices. Adv. Mater. 26, 2699–2703 (2014). 24639376 10.1002/adma.201304681

[b18] Kirz J. Phase zone plates for x rays and the extreme uv. J. Opt. Soc. Am. 64, 301–309 (1974).

[b19] Gu M. Advanced Optical Imaging Theory Springer (2000).

[b20] Vazquez R. M., Eaton S. M., Ramponi R., Cerullo G. & Osellame R. Fabrication of binary Fresnel lenses in PMMA by femtosecond laser surface ablation. Opt. Express 19, 11597–11604 (2011). 21716392 10.1364/OE.19.011597

[b21] Goodman J. W. Introduction to Fourier Optics McGraw-Hill (1968).

[b22] Tao Y. *et al.* Localized insulator-conductor transformation of graphene oxide thin films via focused laser beam irradiation. Appl. Phys. A 106, 523–531 (2012).

[b23] Smirnov V. A. *et al.* Photoreduction of graphene oxide. High Energy Chem. 45, 57–61 (2011).

[b24] Li X. *et al.* Giant refractive-index modulation by two-photon reduction of fluorescent graphene oxides for multimode optical recording. Sci. Rep. 3, 2819 (2013). 24085266 10.1038/srep02819 PMC3788367

[b25] Li X. *et al.* Athermally photoreduced graphene oxides for three-dimensional holographic images. Nat. Commun. 6, 6984 (2015). 25901676 10.1038/ncomms7984 PMC4421811

[b26] Huang L. *et al.* Dispersionless phase discontinuities for controlling light propagation. Nano Lett. 12, 5750–5755 (2012). 23062196 10.1021/nl303031j

[b27] Pors A., Nielsen M. G., Eriksen R. L. & Bozhevolnyi S. I. Broadband focusing flat mirrors based on plasmonic gradient metasurfaces. Nano Lett. 13, 829–834 (2013). 23343380 10.1021/nl304761m

[b28] Gao H. *et al.* Broadband plasmonic microlenses based on patches of nanoholes. Nano Lett. 10, 4111–4116 (2010). 20839781 10.1021/nl1022892 PMC2955180

[b29] Fernandez-Suarez M. & Ting A. Y. Fluorescent probes for super-resolution imaging in living cells. Nat. Rev. Mol. Cell Biol. 9, 929–943 (2008). 19002208 10.1038/nrm2531

[b30] Qiu L., Liu J. Z., Chang S. L. Y., Wu Y. & Li D. Biomimetic superlastic graphene-based cellular monoliths. Nat. Commun. 3, 1241 (2012). 23212370 10.1038/ncomms2251

[b31] Cumming B. P. *et al.* Adaptive optics enhanced direct laser writing of high refractive index gyroid photonic crystals in chalcogenide glass. Opt. Express 22, 689–698 (2014). 24515028 10.1364/OE.22.000689

