# Supplementary material for: Highly efficient and ultra-broadband graphene oxide ultrathin lenses with three-dimensional subwavelength focusing
Source: Nat Commun. 2015 Sep 22;6:8433. doi: 10.1038/ncomms9433 (PMC4595752; doi:10.1038/ncomms9433)
Supplement: Supplementary Information — Supplementary Figures 1-13, Supplementary Note 1 and Supplementary References [file ncomms9433-s1.pdf]

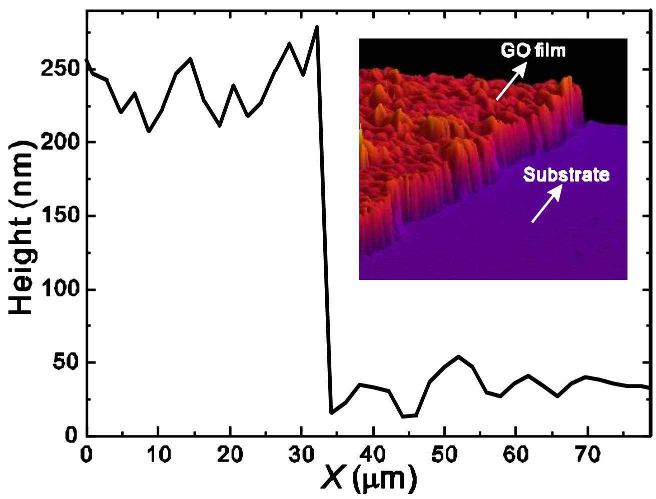

Supplementary Figure 1. **GO thin film thickness characterization**. The thickness of the prepared GO thin film is characterized by using an optical profiler (Bruker ContourGT InMotion). Inset: 3D optical profiler image of the prepared GO film on the substrate showing an averaged GO thickness of ~ 200 nm with an average roughness of < 50 nm. In order to achieve the optimum focusing effect, the thickness of the prepared GO thin film is optimized at 200 nm based on the balanced requirements of the transmission, absorption, thickness variation, and achievable fabrication resolution.

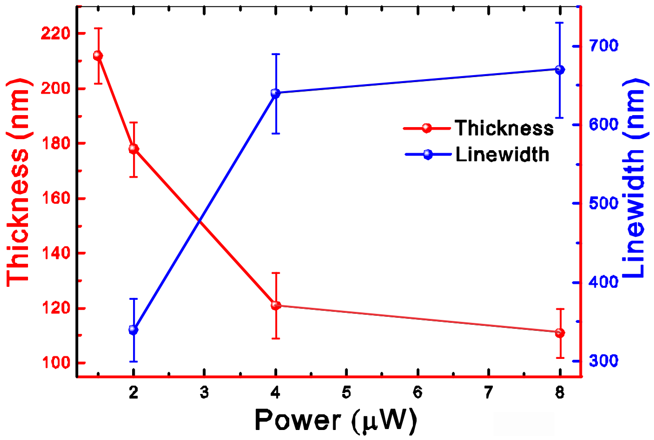

Supplementary Figure 2. **Power dependent film thickness and linewidth.** The dependence of rGO film thickness and linewidth on the power of the fabrication laser is characterized using an ellipsometer (J.A. Woollam M-2000). The linewidths at different laser powers are measured using an optical microscope (Nikon N-STORM) and SEM (RAITH150-TWO). The error bars on both film thickness and linewidth are determined via resampling.

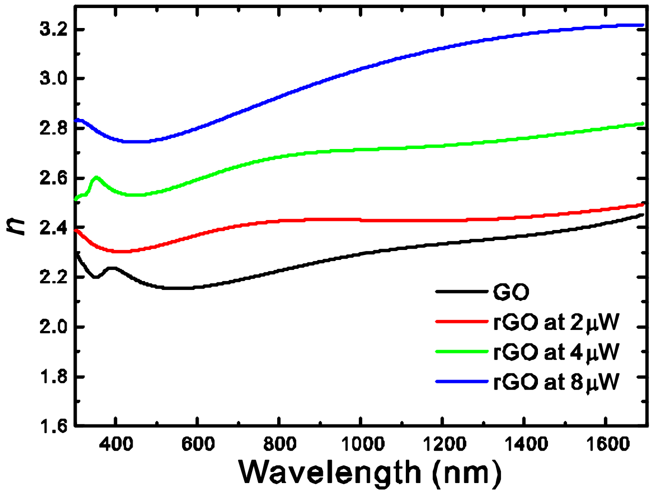

Supplementary Figure 3. **Refractive index of rGO.** The dispersion relations of refractive indices (*n*) of rGO at different laser powers have been measured by using ellipsometry.

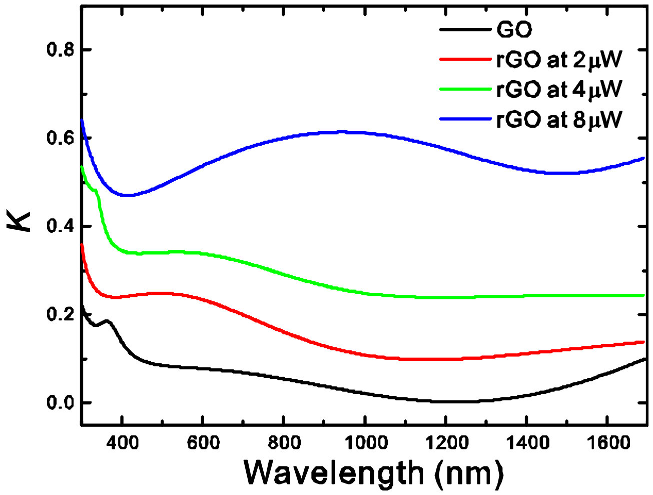

Supplementary Figure 4. **Extinction coefficient of rGO.** The dispersion relations of extinction coefficient (*K*) of rGO at different laser powers have been measured by using ellipsometry.

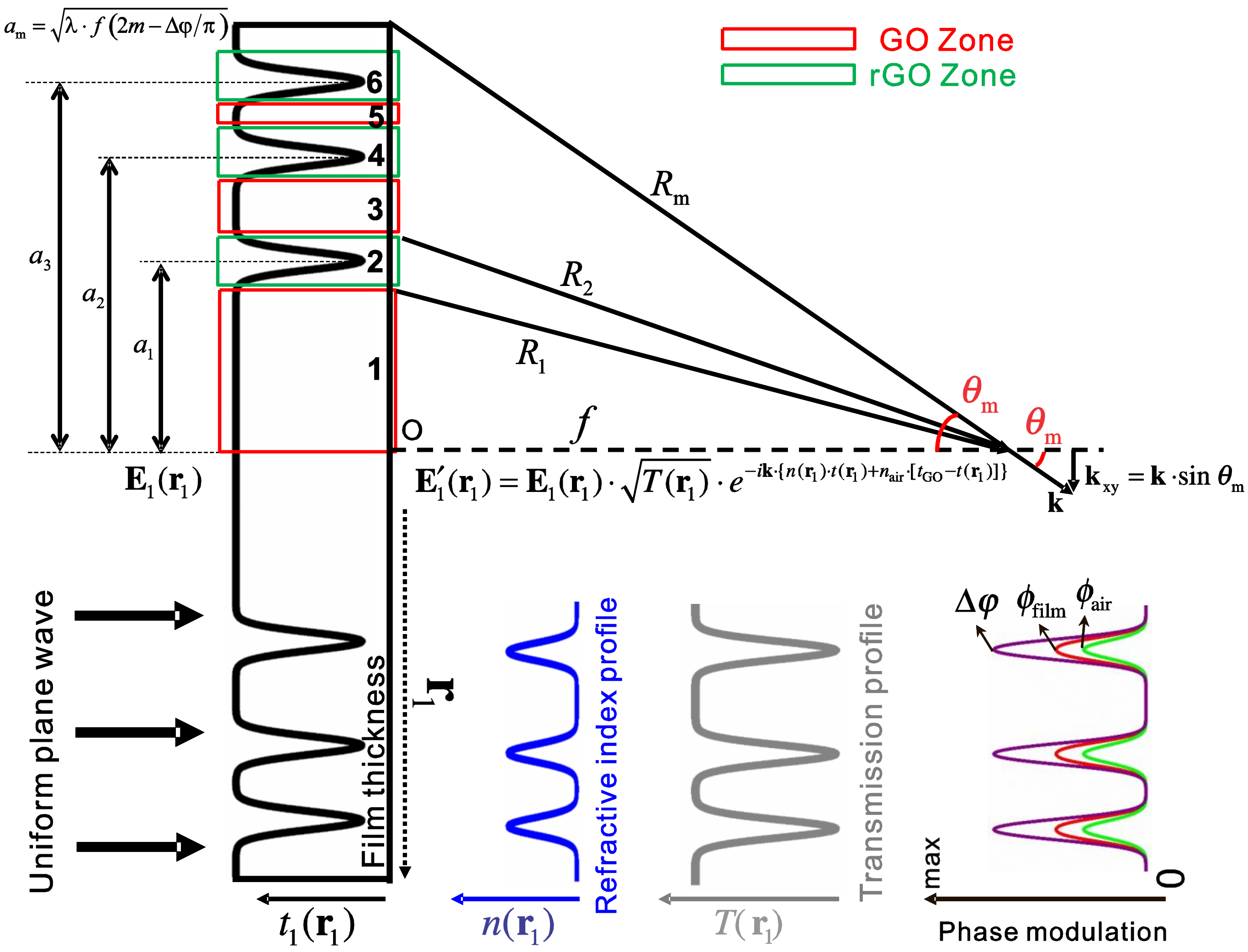

Supplementary Figure 5. **Schematic of the theoretical model of a GO flat lens.** a_m (a_1, a_2 and a_3) is the position of the laser fabricated m^{th} rGO ring. f is the focal length of the GO lens. Six alternating rGO and GO zones are indicated as green and red rectangles, respectively. The Gaussian profiles of film thickness $t(\mathbf{r}_1)$, refractive index $n(\mathbf{r}_1)$ and transmission $T(\mathbf{r}_1)$ have been plotted schematically. The plots of Φ_{film}, Φ_{air} and $\Delta\varphi$ have also been included.

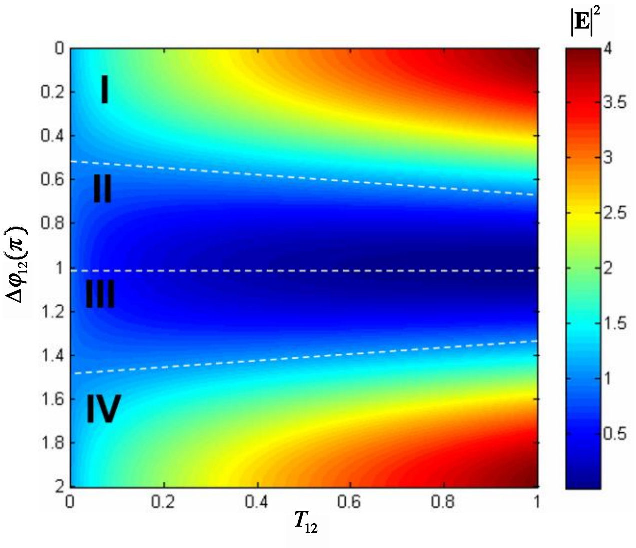

Supplementary Figure 6. **Effects of amplitude and phase modulations.** The interference of two coherent beams is calculated to understand the role of both amplitude and phase modulations.

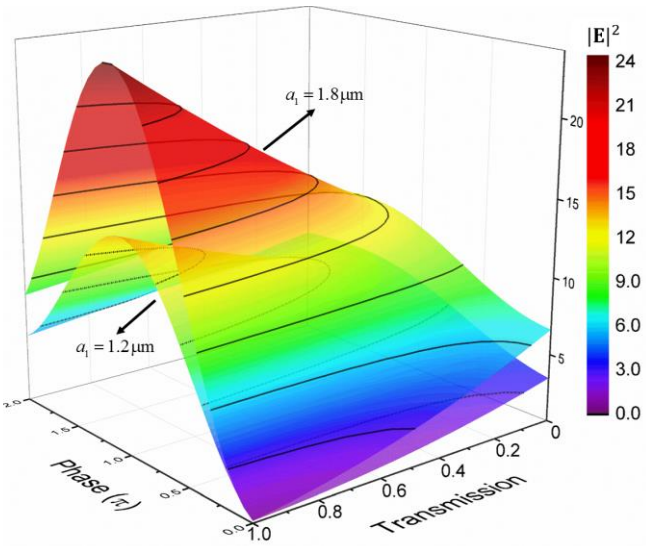

Supplementary Figure 7. **Optimization of amplitude and phase modulations.** Effects of transmission (amplitude) and phase modulations on the GO lens with different a_1.

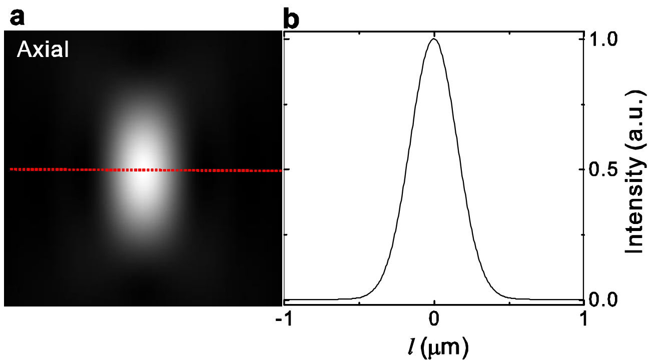

Supplementary Figure 8. **The Gaussian beam profile.** The normalized Gaussian beam profile of the fabrication laser at 800 nm focused by an NA=1.4 objective. (**a**). The intensity distribution of laser beam in the axial direction. (**b**) The cross-section of the intensity distribution along the red dash line in **a**.

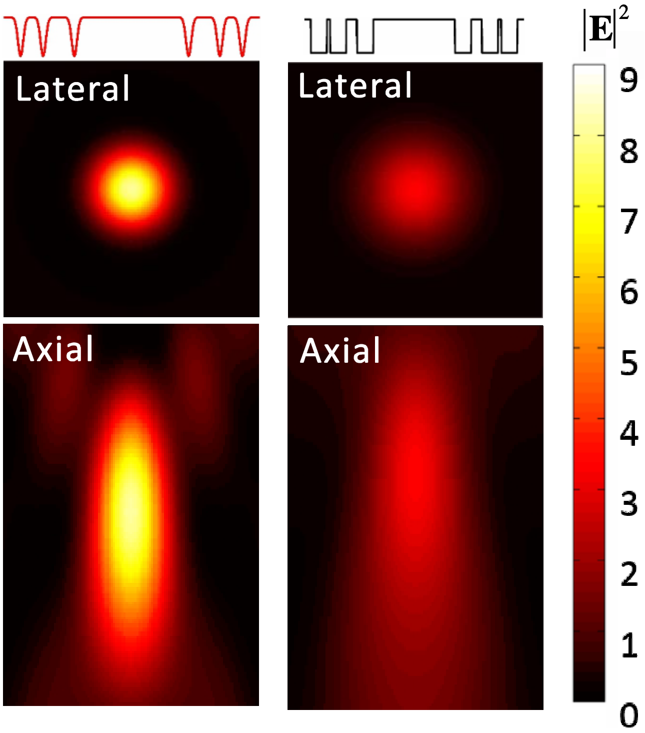

Supplementary Figure 9. **The comparison between Gaussian and rectangular profiles.** The focusing intensity distributions of lenses with Gaussian (left) and rectangular (right) profiles are calculated using our theoretical model respectively. Both lenses are designed with identical geometries (a_1=1.8 μm, N=3) and the illumination wavelength is fixed at 700 nm. The lens with a Gaussian profile shows clearly much stronger focusing intensity with a higher focusing resolution due to the effective direct of the majority of the incident light to the first diffraction order[1].

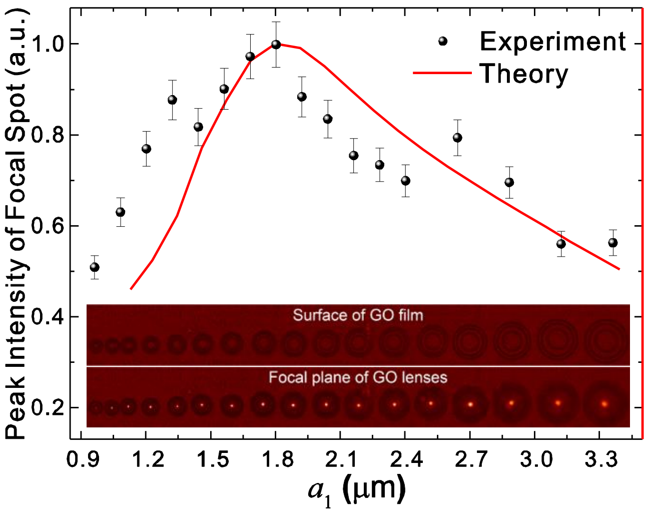

Supplementary Figure 10. **Dependence of focusing intensity on a_1.** The focusing intensity of GO lenses with different a_1 are calculated and measured. For a fixed film thickness, there exists an optimized lens design leading to the strongest focusing intensity. The error bar on intensity is determined via resampling. Inset: Measured images of GO lenses at the surface of GO film (top) and the lens focusing plane (Bottom).

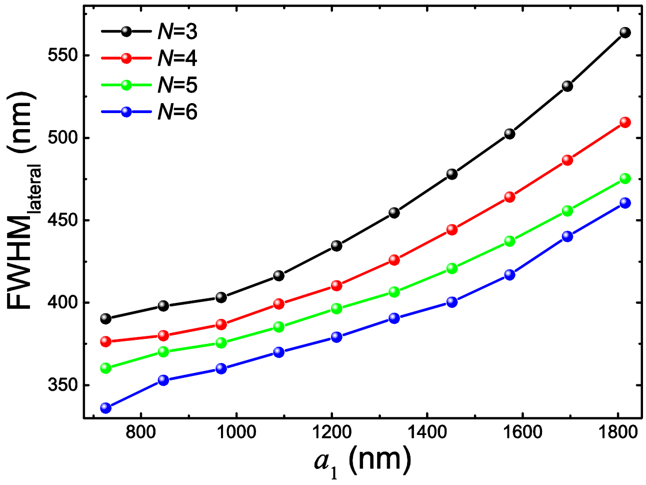

Supplementary Figure 11. **Optimization of focusing resolution.** The dependence of focusing resolution on N and a_1 is calculated. In general, the smaller the first ring size and the larger the total ring number, the better the achievable resolution can be.

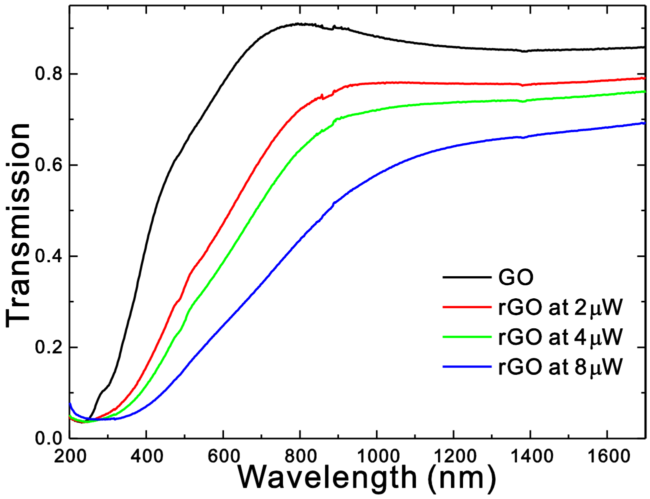

Supplementary Figure 12. **VIS-NIR spectra of rGO films.** The VIS-NIR transmission spectra of rGO at different laser powers are measured using an optical spectrometer (PerkinElmer UV/Vis Spectrophotometer). For increased laser powers, the transmission spectra present similar trends with reduced strength. In the longer wavelength range, the spectra exhibit dispersionless feature, advantageous for ultra-broadband lens design.

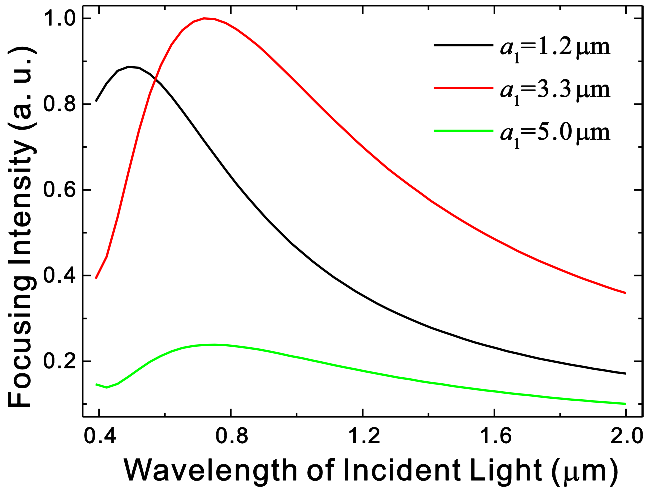

Supplementary Figure 13. **Optimization of broadband focusing intensity.** The dependence of broadband focusing intensity of GO lenses on the first ring size (a_1) is calculated. For each lens with different a_1, an optimal wavelength exists at which the maximum focusing intensity can be achieved. As a result, to achieve a larger operation bandwidth for a single GO lens with maximum focusing intensity, a_1 is selected to be 3.3 μm.

Supplementary Note 1

Analytical Method

The intensity distribution in the focal region of the ultrathin lens can be calculated using the Rayleigh-Sommerfeld diffraction theory[2] based on the Fresnel approximation with a circular symmetry as:

$$\left|\mathbf{E}_2(\mathbf{r}_2,z)\right| = \frac{i2\pi}{\lambda z}\exp(-i\left|\mathbf{k}\right|z)\exp(-\frac{i\left|\mathbf{k}\right|\cdot\mathbf{r}_2^2}{2z})\int_0^{+\infty}\left|\mathbf{E}_1(\mathbf{r}_1)\right|\exp(-\frac{i\left|\mathbf{k}\right|\cdot\mathbf{r}_1^2}{2z})J_0(\frac{\left|\mathbf{k}\right|\cdot\left|\mathbf{r}_1\right|\cdot\left|\mathbf{r}_2\right|}{z})\left|\mathbf{r}_1\right|d\mathbf{r}_1 \quad (1)$$

where the subscripts ‘1’ and ‘2’ indicate the parameters in the lens plane and in the focal region, respectively. As a result, the field distributions in the focal region ($\mathbf{E}_2$) at different lateral ($\mathbf{r}_2$) and axial (z) positions can be calculated by integrating the field distribution ($\mathbf{E}_1(\mathbf{r}_1)$) over the entire lens plane. One example showing here is a GO lens design with three rGO zones, as shown in Supplementary Figure 5.

When a uniform plane wave ($\mathbf{E}_1(\mathbf{r}_1)=1$) impinges the GO lens, part of the beam is absorbed and refracted by the rGO zones, experiencing substantial amplitude as well as phase modulations. The other part of the beam propagates through the GO zones only experiencing ignorable amplitude modulation. The modulated E-field becomes:

$$\mathbf{E}_1'(\mathbf{r}_1) = \mathbf{E}_1(\mathbf{r}_1)\cdot\sqrt{T(\mathbf{r}_1)}\cdot e^{-i\mathbf{k}(\Phi_{\text{film}}+\Phi_{\text{air}})} \quad (2)$$

where $T(\mathbf{r}_1)$ is the transmission distribution, which can be calculated using the Beer-Lambert equation[3]:

$$T(\mathbf{r}_1) = e^{-\alpha(\mathbf{r}_1)\cdot t(\mathbf{r}_1)} \quad (3)$$

where $\alpha(\mathbf{r}_1)$ is the absorption coefficient which can be calculated from extinction coefficient $K(\mathbf{r}_1)$ through $\alpha(\mathbf{r}_1)=4\pi\times K(\mathbf{r}_1)/\lambda$. Φ_{film} ($=n(\mathbf{r}_1)t(\mathbf{r}_1)$) and Φ_{air} ($=n_{\text{air}}[t_{\text{GO}}-t(\mathbf{r}_1)]$) are the phase modulations provided by the film and the air, respectively. Meanwhile, the modulated

refractive index $n(\mathbf{r}_1)$, thickness $t(\mathbf{r}_1)$ and extinction coefficient $K(\mathbf{r}_1)$ due to the laser photo-reduction can be formulated as:

$$\begin{cases} n(\mathbf{r}_1) = n_{\mathrm{GO}} + \Delta n \cdot M(\mathbf{r}_1) \\ t(\mathbf{r}_1) = t_{\mathrm{GO}} + \Delta t \cdot M(\mathbf{r}_1) \\ K(\mathbf{r}_1) = K_{\mathrm{GO}} + \Delta K \cdot M(\mathbf{r}_1) \end{cases} \tag{4}$$

Here n_{GO}=2.2 is the refractive index of the GO film at 700 nm. t_{GO} = 200 nm is the thickness of the GO film. K_{GO} = 0.07 is the extinction coefficient of GO at 700 nm. All these parameters are measured experimentally. The modulation function M is expressed as:

$$M(\mathbf{r}_1) = C\sum_{m=1}^{N} e^{\frac{(\mathbf{r}_1 - a_{\mathrm{m}})^2}{2w^2}} \tag{5}$$

where C is a constant depending on the femtosecond laser power. a_{m} is the position of mth rGO zone, and N is the total number of rGO zones (N=3 in this design). Note that the modulation function M shows a Gaussian shape governed by the intensity distribution of the laser focus, with w controlling the full width at a half maximum (FWHM). To this end, we have successfully applied the Gaussian profiles of material properties as well as the geometries of the GO lens into the analytical model, which is ready to calculate the performance of the GO lens made of materials with different physical properties at various geometries and is used all through this paper.

Effects of amplitude and phase modulations.

To understand the role of amplitude and phase modulations, the interference of two coherent beams with certain phase and amplitude modulations is studied firstly. Assuming that the electric fields of beam 1 and beam 2 are:

$$\begin{cases} \mathbf{E}_1 = A_1 \cdot e^{-i\varphi_1} \\ \mathbf{E}_2 = \sqrt{T_{12}} \cdot A_1 \cdot e^{-i(\varphi_1 + \Delta\varphi_{12})} \end{cases}$$

where T_{12} and $\Delta\varphi_{12}$ correspond to the amplitude and phase modulations. As a result, the intensity of the interference beam can be calculated directly via $|\mathbf{E}|^2 = |\mathbf{E}_1 + \mathbf{E}_2|^2$. Therefore, the dependence of the interference intensity on the phase and amplitude modulations can be calculated, as shown in Supplementary Figure 6. We can see clearly that the largest intensity occurs when the phase modulation ($\Delta\varphi_{12}$) is either zero or 2π with no amplitude modulation (T_{12}=1), which is well known as the constructive interference condition with $|\mathbf{E}|^2 = 4|\mathbf{E}_1|^2$. Moreover, four regions with different roles of amplitude and phase modulations can be distinguished, as indicated in Supplementary Figure 6. In regions I and III, increase of phase modulation reduces the interference intensity, whereas the interference intensity increases with the phase modulation in regions II and IV. On the other hand, in regions I and IV, larger amplitude modulation deteriorates the interference intensity. However, in regions II and III it is preferred to have larger amplitude modulation.

As a result, to show the effects of both phase and amplitude modulations in a general lens design without limiting to any particular case, numerical calculations were applied to map over the complete phase modulation (0~2π) and amplitude modulation (0~1), as shown in Supplementary Figure 7. One can see clearly that different roles of amplitude and phase modulations can be distinguished, which shows similar behaviour as two beam interference (Supplementary Figure 6). Moreover, the unique surface contour shows the dependence of the lens performance (e. g. lens peak focusing intensity normalized to the incident beam intensity in Supplementary Figure 7) on the phase and amplitude modulations. As a result, given the attainable material properties, both the amplitude and phase modulations, and the geometry of the lens can be optimized to achieve the best performance.

Compared to the conventional amplitude type Fresnel zone plate, more light is transmitted for our GO lens. The predicated maximum efficiency reaches >50%, which breaks the theoretical limitation of focusing efficiency of amplitude-type Fresnel zone plate (~10%)[3].

Although 2π phase shift is not achieved between the adjacent GO and rGO zones, the Gaussian profile of each rGO boosts the overall GO lens performance over phase type Fresnel lens in subwavelength 3D resolution, ultrathin lens thickness and smaller lens sizes with a focusing efficiency (>50%) exceeding the theoretical limitations of phase type Fresnel lens (40%).

Derivation of optimized ring position a_m

For our GO lens, the focusing is the interference of light passing through the GO and rGO zones, which provide phase and amplitude modulations. The phase modulation between the adjacent GO and rGO zones (only zone 1 and zone 2 are shown here) can be expressed as:

$$\Delta\phi = \frac{2\pi}{\lambda} \cdot (R_2 - R_1) + \Delta\varphi \,, \tag{6}$$

where $\Delta\varphi$ is the phase modulation between GO and rGO zones. Given the fact that the phase modulation within one rGO zone is comparatively weak, it is physically sound to approximate the phase modulation $\Delta\varphi$ as a constant across each rGO zone, which is taken as the average of the Gaussian function. To guarantee a constructive interference between all the GO zones and rGO zones, $\Delta\phi$ should be fixed at 2π, corresponding to constructive interference. As a result, we can get:

$$(R_m - f) = m\lambda - \lambda \cdot \frac{\Delta\varphi}{2\pi} \tag{7}$$

where f is the focal length of GO lens, as indicated in Supplementary Figure 5. Therefore, by solving the Supplementary Equation 7 using the relation $R_m^2 = a_m^2 + f^2$, we can easily obtain that:

$$a_m = \sqrt{\lambda \cdot f(2m - \Delta\varphi / \pi)} \tag{8}$$

During the laser-induced reduction process of GO, three physical properties (film thickness, refractive index and extinction coefficient) are correlated and all dependent on the reduction

extent, which is eventually controlled by the laser power. Therefore, for a given phase modulation, the corresponding amplitude modulation is determined. As a result, we are able to consider both the amplitude and phase modulations at the same time by only including the phase modulation factor in the equation (Supplementary Equation 8) for the lens design.

The amplitude and phase modulations were optimized according to the following two criteria. 1): Maximizing the constructive interference between all the GO zones and all the rGO zones. 2): Minimizing the destructive interference between the adjacent GO and rGO zones. In this way, it is possible to achieve the best focusing/interference condition for a given phase/amplitude modulation to eventually optimize both the focal spot size and the focusing efficiency.

Derivation of wavelength independence focusing

The effective wave vector along the lateral direction which can be formulated as:

$$k_{\mathrm{xy}} = \mathbf{k} \cdot \sin\theta_{\mathrm{m}} \tag{9}$$

Here θ_{m} is the diffraction angle between the wave vector and the optical axis (See Supplementary Figure 5) of the light from the mth zone (a_{m}), which can be calculated by the diffraction equation[3] as:

$$\sin\theta_{\mathrm{m}} = \lambda / (a_{\mathrm{m}} - a_{\mathrm{m-1}}) \tag{10}$$

By substituting Supplementary Equation 10 into Equation 9, one can obtain:

$$k_{\mathrm{xy}} = 2\pi / (a_{\mathrm{m+1}} - a_{\mathrm{m}}) \tag{11}$$

which is independent of the incident wavelength.

Supplementary References

1. Goodman, J. W. Introduction to Fourier Optics (McGraw-Hill, New York, 1968).

2. Gu, M. *Advanced Optical Imaging Theory*. (Springer, Berlin Heidelberg, 2000).

3. Zheng, X., Jia, B., Chen, X. & Gu, M. In situ third-order nonlinear responses during laser reduction of graphene oxide thin films towards on-chip nonlinear photonic devices. *Adv. Mater.* **26**, 2699-2703 (2014).

4. Hecht, E. *Optics* (Addison-Wesley, 2002).
